# Chest wall tuberculosis - A clinical and imaging experience

**DOI:** 10.4103/0971-3026.76051

**Published:** 2011

**Authors:** Shabnam Bhandari Grover, Meghna Jain, Shifali Dumeer, Nanda Sirari, Manish Bansal, Deepak Badgujar

**Affiliations:** Department of Radiology and Imaging, Vardhman Mahavir Medical College and Safdarjang Hospital, New Delhi, India; 1 Department of Orthopedic Surgery, Vardhman Mahavir Medical College and Safdarjang Hospital, New Delhi, India

## Abstract

**Aims:**

Tuberculous infection of the thoracic cage is rare and is difficult to discern clinically or on radiographs. This study aims to describe the common sites and the imaging appearances of chest wall tuberculosis.

**Materials and Methods:**

A retrospective review of the clinical and imaging records of 12 confirmed cases of thoracic cage tuberculosis (excluding that of the spine), seen over the last 7 years, was performed. Imaging studies available included radiographs, ultrasonographies (USGs), and computed tomography (CT) scans. Pathological confirmation was obtained in all cases.

**Results:**

All patients had clinical signs and symptoms localized to the site of involvement, whether it was the sternum, sternoclavicular joints, or ribs. CT scan revealed sternal destruction in three patients and osteolytic lesions with sclerosis of the articular surfaces of the sternoclavicular joints in two patients. In five patients with rib lesions, USG elegantly demonstrated the bone destruction underlying the cold abscess. All cases were confirmed to be of tuberculous origin by pathology studies of the aspirated/curetted material, obtained by CT / USG guidance.

**Conclusions:**

Tuberculous etiology should be considered for patients presenting with atypical sites of skeletal inflammation. CT scan plays an important role in the evaluation of these patients. However, the use of USG for demonstrating rib destruction in a chest wall cold abscess has so far been under-emphasized, as has been the role of CT and USG guided aspiration in confirming the aetiology.

## Introduction

Chest wall involvement is an uncommon manifestation of tuberculosis. Tuberculous localization in the thoracic cage is rare and difficult to discern on radiographs.[1] This entity has been reported so far in only a few series or as isolated case reports.[2–11] We report our experience of 12 immune-competent patients with chest wall tuberculosis involving the sternum, sternoclavicular joints, and ribs; spinal tuberculosis cases were excluded. We describe the radiographic, USG, and CT scan findings in these patients. The majority of earlier investigators have focused on the use of CT scan and MRI in the diagnosis of chest wall tuberculosis. We have also shown the vital role of USG in the diagnosis of rib destruction and associated cold abscess along with importance of CT and USG guided aspiration in confirming the aetiology.

## Materials and Methods

We retrospectively reviewed the clinical and imaging records of 12 patients with chest wall tuberculosis. Patients with isolated dorsal spine infection were excluded.

Imaging studies available included radiographs, USGs, and CT scans. Chest radiographs and radiographs of clinically involved sites, were available in all patients. In patients with clinical evidence of chest wall abscess, USG was performed using a high-frequency transducer (Sonoline Adara, Siemens, Japan). CT scan of the chest was performed in all patients with pain and swelling over the sternum and sternoclavicular joint.

Other sites of involvement were excluded by clinical examination, skeletal survey, and USG. Pathology confirmation was obtained in all patients, using CT/ USG guided aspiration cytology in eight and surgical exploration/curettage in four cases.

## Results

All patients were immunocompetent. The clinical features, laboratory findings, and imaging observations are summarized and serialized as patient numbers in Table 1.

**Table 1:** Summary of clinical, radiological (and imaging), and pathology data in 12 patients of chest wall tuberculosis

No.	Patient age (yr)/ sex	Clinical information	Duration of symptoms	Radiographic findings	Ultra sound findings	CT findings	Other sites of involvement	Confirmation of tuberculosis by
1.	26 / M	Pain in back, swelling over sternum, fever, high ESR, Mtx +	6 months	Chest-paravertebral mass extending from D5-D12.	Not referred for	Radiographic findings pertaining to spine confirmed.	Spine	CT guided aspiration from paravertebral Abscess. Langhans giant cell, epitheloid granuloma and Lymphocytes seen
				Dorsal spine-destruction of D9-D10, with loss of disc space, pre-& paravertebral abscess present		Erosion with adjacent sclerosis seen at Rt sternal margin with adjoining soft tissue thickening & enhancement		granuloma and
2.	25 / M	Painless ulcerated swelling over sternum, discharging sinus	4 months	Lateral view sternum- lytic lesion in body of sternum with associated soft tissue swelling	Not referred for	Destruction with sclerosis body of sternum with presternal soft tissue swelling & skin ulceration		Debridement & Curettage sternum, AFB seen
3.	7 / M	Pain, swelling over sternum, fever	2 months	Chest - Mediastinal widening, LUZ infiltrates	Not referred for	Destruction with sclerosis body of sternum	Lung, Mediastinum	CT guided aspiration, AFB seen
4.	53 / F	Pain, swelling overlying Rt. sternoclavicular jt., fever, high ESR	6 months	Chest- parenchymal infiltration seen bilaterally	Not referred for	Expansion with multiple erosions medial end Rt Clavicle,	Lung	Surgical curettage, epitheloid granuloma and caseous necrosis seen
						Parenchymal infiltrates in bilateral upper zones		
5.	23 / F	Pain, swelling overlying Lt. sternoclavicular joint, fever, high ESR, Mtx+, sputum for AFB+	2 months	Chest-Mediastinal widening, LUZ Infiltrates	Not referred for	Mediastinal adenopathy, Sclerosis with minimal irregularity of articular surface Lt. clavicle, Parenchymal infiltrates LUZ	Lung, Mediastinum	CT guided aspiration, Langhans giant cell and lymphocytes seen
				Oblique view Lt Sternoclavicular Joint - dense sclerosis of articular surfaces.				
6.	11 / F	Painless swelling Lt ant. chest wall, fever, high ESR, Mtx+	1 Month	Erosion anterior end of Lt 4th rib, parenchymal infiltration Lt upper zone.,	Hypoechoic collection 2×2cm overlying Lt. 4th rib anteriorly and rib destruction present	Not done	Lung	USG guided aspiration, AFB seen
7.	2/M	Painless swelling Rt scapular & interscapular region, fever, high ESR, Mtx+	2 Weeks	Erosion post end of Rt. 3^rd^ rib, Mediastinal lymph nodes, Parenchymal infiltration bilaterally	Hypoechoic collection Rt scapular region measuring 2.9×1.5cm with internal heterogenous echotexture. No rib destruction seen	Not done	Lung. Mediastinum	USG guided aspiration, AFB Seen
8.	9/F	Pain lower back, fever, paraparesis, non healing ulcer scalp, high ESR, Mtx+	1 year	Chest-Expansile destruction & sclerosis post. End Lt 10^th^ rib	Not referred for by the treating clinician: directly proceeded for surgical curettage	Not done	Skull, Sacroiliac jt.	Debridement & curettage of skull & rib lesions, AFB epitheloid granulomas with caseous necrosis seen.
				Skull-irregular lytic lesion with marginal sclerosis Rt. Parietal bone				
				Pelvis-sclerosis Rt. SI jt.				
9.	8 / F	Pain, fever, cough, swelling Rt. Forearm, high ESR, Mtx+	3 Months	Chest - Mediastinal widening, pleural effusion, sclerosis & widening ant end of Lt 3^rd^ and 4^th^ ribs	Not referred for	Not done	Ulna, Pleura	Curettage from ulna, Langhans giant cell, epitheloid granuloma, lymphocytes seen
				X ray B/L forearm- right ulnar osteomyelitis with profound periosteal reaction				
10.	25 / F	Pain, lump in medial lower quadrant Lt breast, high ESR, sputum for AFB+	3 Months	Chest- left hilar prominence, parenchymal infiltration RUZ-MZ, erosion superior margin of anterior end Lt 4th rib	Hypoechoic collection in lower outer quadrant of Lt breast (3×1.5 cm), destruction of underlying rib with free fragments within the abscess	Not done	Lung, Mediastinum	USG guided aspiration of retro-mammary abscess, AFB seen
11.	23 / M	Pain, swelling Rt. Chest wall anteriorly, fever, high ESR Mtx+, sub- acute intestinal Obstruction	1 Month	Normal	Hypoechoic collection, measuring 4×1.5cm located in Rt ant. chest wall, ascites present	Not done	Peritoneum	USG guided aspiration of abscess: Langhans giant cell, epitheloid granulomas, lymphocytes seen
12.	9 / M	pain, swelling Lt. chest wall posteriorly, fever, high ESR	2 Months	Chest-Mediastinal widening, parenchymal infiltration Lt para-hilar region, Lt pleural effusion	Hypoechoic collection 5×2 cm with echogenic walls & low level internal echoes overlying Lt. kidney, Lt. pleural effusion	Not done	Pleura	USG guided aspiration of abscess: epitheloid granuloma, Langhans giant cell and lymphocytes seen

Mtx+, Montoux positive; Lt, left; Ant, anterior; AFB, acid fast bacillus; post, posterior; Rt, right; LUZ, left upper zone; RUZ, right upper zone; MZ, middle zone; B/L, bilateral

**Figure 1 (A–C):**
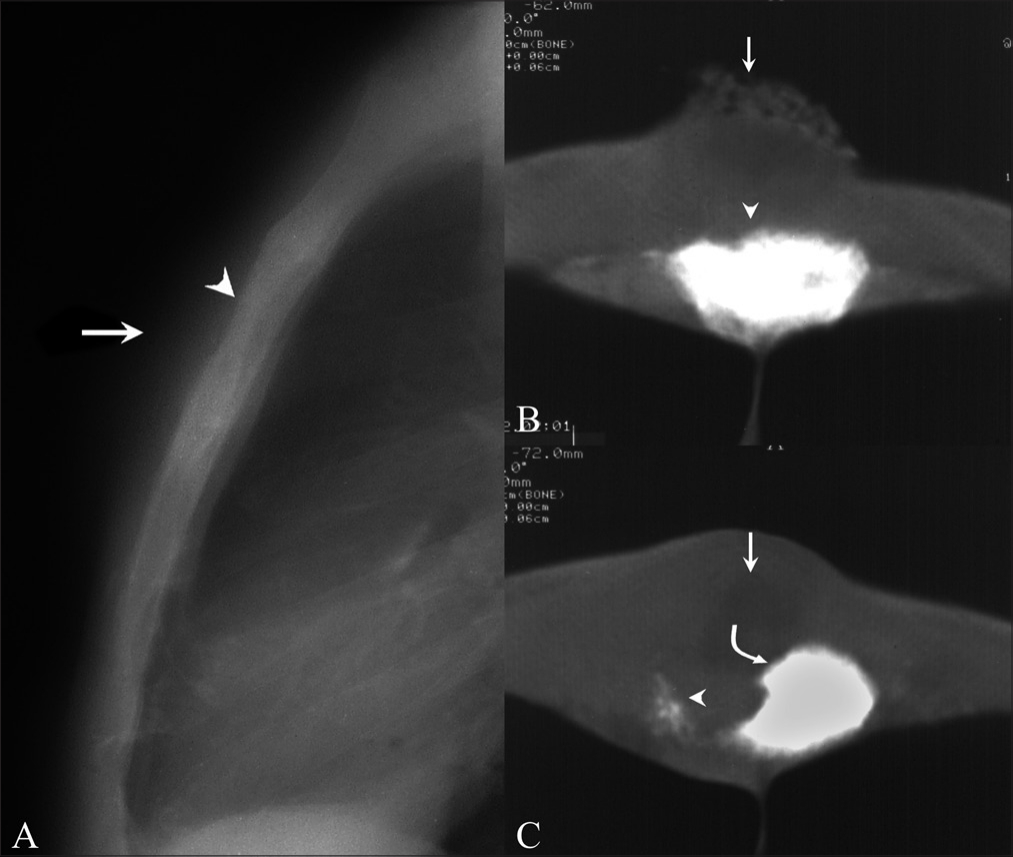
Sternal tuberculosis – patient 2. Lateral radiograph (A) of the sternum shows an osteolytic lesion (arrowhead) with overlying soft tissue swelling (arrow). CT scans (B, C) show dense sclerosis of the sternal body (arrowhead in B) associated with a large skin ulcer (arrow in B) with osteolysis (arrowhead in C) more caudally, with sclerosis of the remaining fragment (curved arrow in C) and an adjoining soft tissue abscess (arrow in C)

**Figure 2 (A–C):**
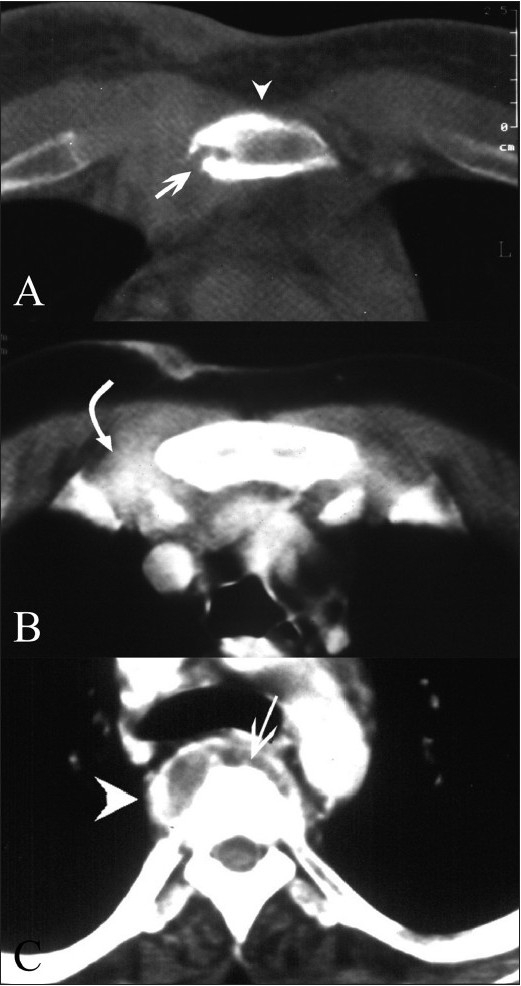
Sternal tuberculosis – patient 1. Contrast-enhanced CT scans show erosion (arrow) and sclerosis (arrowhead) of the sternum. The adjoining right parasternal soft tissue shows thickening with dense enhancement (curved arrow). Vertebral destruction (arrow in C) and a paravertebral abscess (arrowhead in C) are also noted

Three patients (patients 1, 2, and 3) presented with a sternal swelling. Radiographs revealed an osteolytic lesion of the sternum with adjacent soft tissue swelling in patient number 2 [Figure 1A]. No sternal changes were radiographically discernable in the other two. CT scan of these three patients revealed sternal destruction and sclerosis with associated soft tissue involvement [Figures 1B and C and 2A–C]. Patients 1 and 3 underwent CT guided aspiration for confirmation of the diagnosis, which revealed caseous material with acid-fast bacilli (AFB) and giant cells. Patient 2 underwent debridement and curettage, which revealed AFB and epitheloid granulomas with giant cells.

**Figure 3 (A, B):**
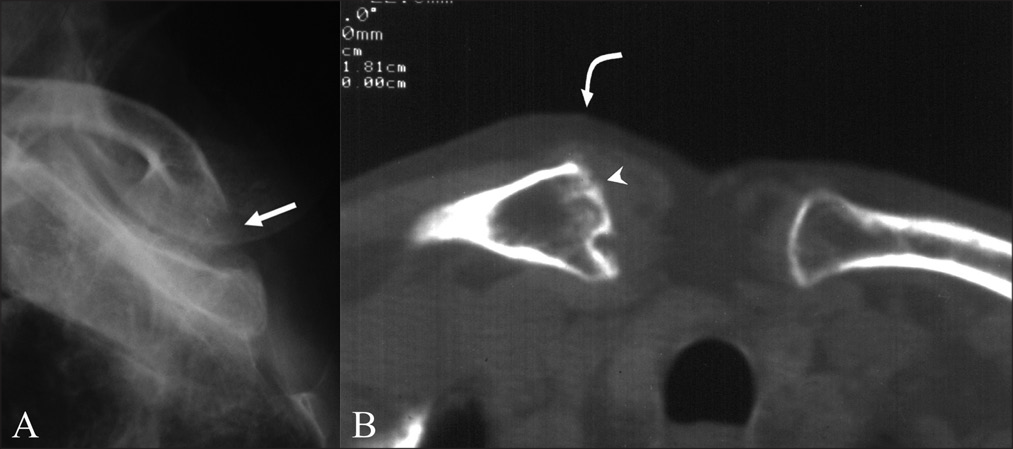
Sterno clavicular tuberculosis – patient 4. Oblique radiograph of the sternoclavicular joint (A) shows an osteolytic lesion (arrow) on the medial articular surface of the right clavicle. CT scan of the sternoclavicular joint (B) shows multiple erosions and expansion of the medial end of the right clavicle (arrow head). Adjacent soft tissue thickening is seen (curved arrow)

Two patients presented with symptoms of sternoclavicular joint involvement (patients 4 and 5). Osteolytic lesions of the clavicular margins were radiographically evident in patient 4 [Figure 3A]. Radiographs showed soft tissue swelling and dense sclerosis of the articular surfaces in patient 5. CT scan in patient 4 revealed expansion of the medial end of the clavicle and erosions of its articular surface [Figure 3B] with dense sclerosis of the articular surfaces in patient 5. The diagnosis was confirmed on CT guided aspiration in one patient and by surgical curettage in the other, with both cases revealing epitheloid granulomas and giant cells.

**Figure 4 (A–C):**
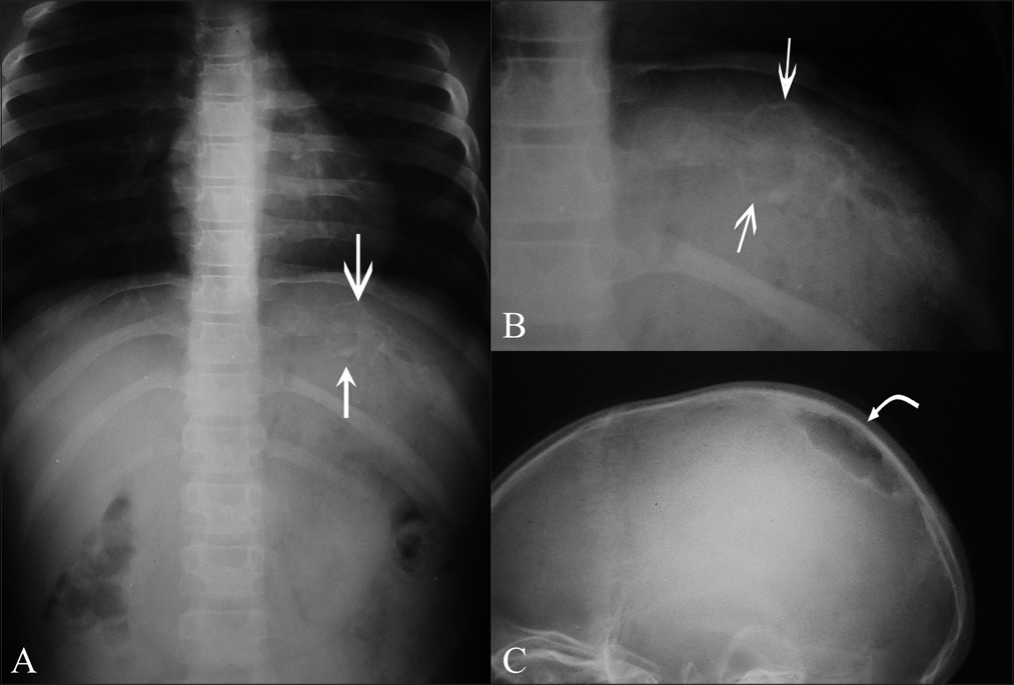
Rib tuberculosis – patient 8. Frontal radiographs of the ribs (A, B) show an expansile osteolytic lesion with sclerosis involving the posterior end of the left 10^th^ rib (arrows). Lateral skull radiograph (C) of the same patient shows a sharply marginated osteolytic lesion, with marginal sclerosis in the right parietal bone (curved arrow)

Radiographs were obtained in seven patients clinically suspected to have rib involvement (patients 6-12). Patients 6, 7, and 10 showed rib erosions. Patient 8 had mixed osteolytic and sclerotic changes in the left 10th rib along with chronic osteomyelitis of the skull vault [Figure 4A–C]. Patient 9 had sclerosis and widening of the left third and fourth ribs, with ipsilateral empyema. No rib changes were discernable in patients 11 and 12.

**Figure 5:**
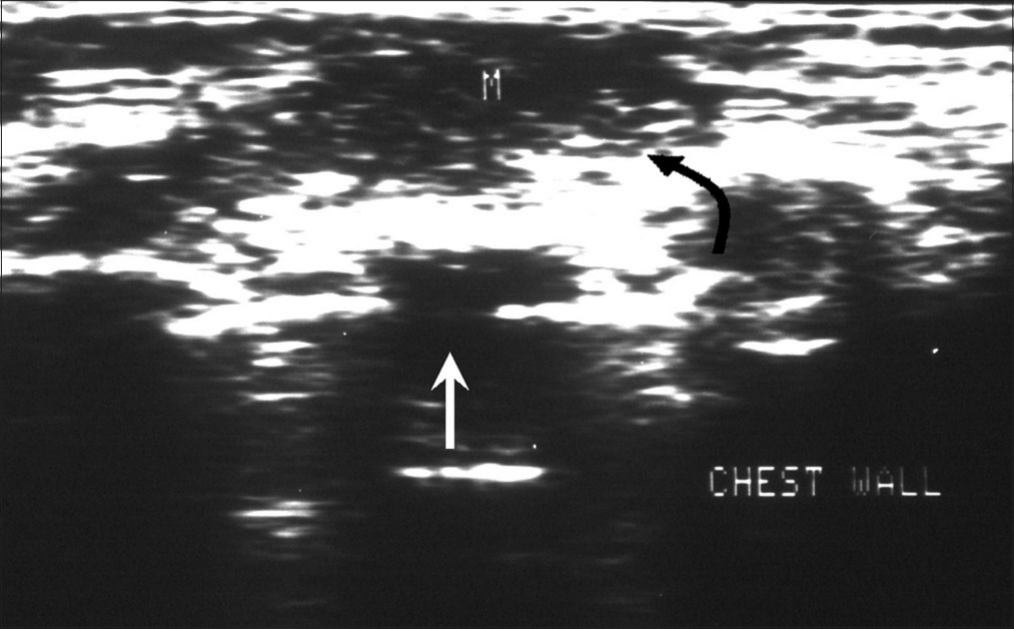
Rib tuberculosis – patient 6. Ultrasonography of the chest wall shows rib destruction (white arrow) with an associated hypoechoic abscess (“M,” curved black arrow)

**Figure 6:**
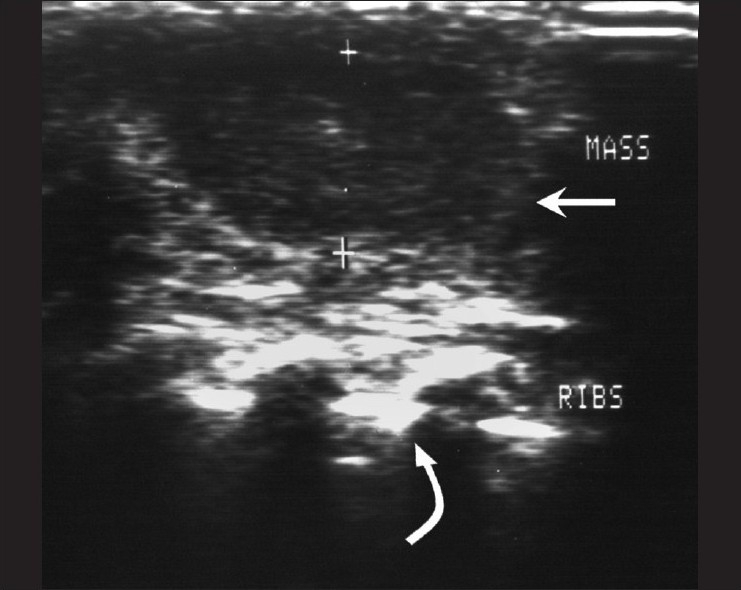
Rib tuberculosis – patient 7. Ultrasonography of the chest wall shows a hypoechoic collection with internal echoes (arrow) overlying the ribs (curved arrow)

**Figure 7:**
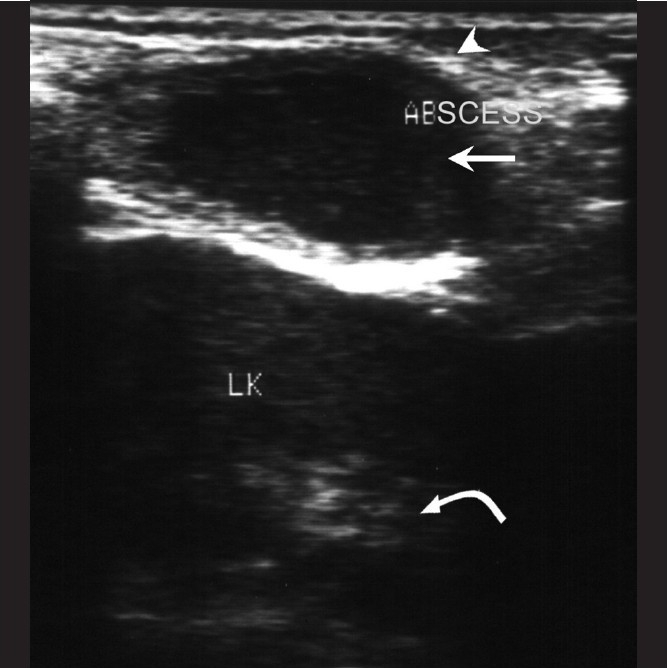
Rib tuberculosis – patient 12. Ultrasonography of the lower chest wall shows a hypoechoic collection (arrow) with echogenic walls (arrow head) overlying the left kidney (curved arrow)

**Figure 8:**
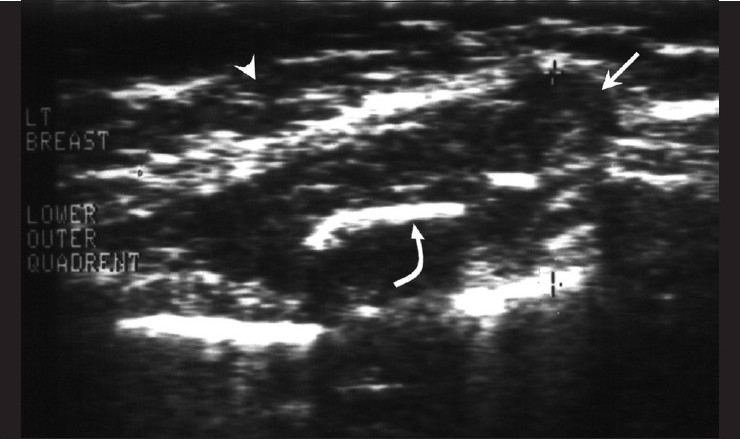
Rib tuberculosis – patient 10. Ultrasonography of the lateral quadrant of the left breast (arrow head) shows a hypoechoic collection (arrow) within which echogenic bone fragments of destroyed ribs (curved arrow) are seen.

USG was performed in five patients with suspected rib involvement (patients 6, 7, and 10-12). The scans revealed rib destruction and cold abscess in patients 6, 7, 11, and 12 [Figures 5–7]. The internal echotexture of the abscesses varied from uniformly low echogenicity to heterogeneous echogenic contents. Patient 10 presented with a left breast lump and USG revealed a retro-mammary abscess in the lower outer quadrant within which echogenic bone fragments of the destroyed rib were seen [Figure 8].

USG-guided aspiration in patients 6, 7, and 10-12 revealed AFB and caseating material, confirming the diagnosis of tuberculosis. Surgical curettage from the skull and the left 10th rib in patient 8 and from the ulna in patient 9 showed Langhans giant cells and lymphocytes, confirming the tuberculous etiology.

## Discussion

Tuberculosis of the chest wall constitutes 1-5% of all cases of musculoskeletal tuberculosis, which in turn represents between 1 and 2% of the total cases of tuberculosis.[11] Common sites of skeletal tuberculosis are spine, hip joint, knee joint, foot, elbow, hand, and shoulders.[1] The sternum, ribs, and the sternoclavicular joints are uncommonly affected.[2,3,5] Tuberculosis of the ribs constitutes 2% and that of the sternum and sternoclavicular joints about 1-2% of the total cases of musculoskeletal tuberculosis.[1,2] The incidence is not expected to stay so low in the near future due to the emergence of multidrug-resistant strains and the rapid increase in the number of immunocompromised patients.[4]

Some investigators believe that chest wall tuberculosis occurs by reactivation of latent foci formed during hematogenous or lymphatic dissemination of primary tuberculosis, while others opine that it occurs by direct extension from contiguous lung/pleura.[3–5] We believe that the former route (hematogenous/lymphatic) is more likely, as only one patient in our series had rib involvement with contiguous empyema.

Radiographic and CT scan findings of tuberculous sternal osteomyelitis have been reported as bone loss, which is relatively frequent, and rarely as sclerosis.[5] In our patients, bone destruction was evident radiographically in only one of the three patients with sternal tuberculosis. CT scan detected it in the other two patients in whom the findings were mainly of sternal destruction and sclerosis with associated abscess. Other investigators who have used MRI have reported bright bone and bone marrow signal on T2W images, with enhancement of bone marrow along with abnormal, enhancing peristernal soft tissue in contrast enhanced studies.[5]

The CT features of sternoclavicular tuberculosis as described in the literature include bone destruction, soft tissue masses crossing fascial planes, with abscess and calcification as well as underlying pleuro-parenchymal tubercular involvement.[2] Similar findings were seen in our patients, and these were better documented on the CT scan. Therefore, the role of CT scan in the evaluation of the sternum and the sternoclavicular joint is evident. Presently, other authors also recommend CT scan for evaluation of the sternum and sternoclavicular joint.[2,3,5,11] MRI may also help, and shows marrow changes in the sternum and clavicle, hypointense on T1W and hyperintense on T2W sequences.[2]

The radiographic and CT scan features of rib tuberculosis have been described in the literature as rib erosions and destruction, with adjacent abscess formation.[3,7,8,11] Similar findings were obtained in our study. There is a paucity of reports highlighting the use of USG as a cost-effective and useful modality to assess rib destruction and associated soft tissue abscess. In our study, we have highlighted the role of USG in diagnosing osteolytic rib lesions. USG elegantly demonstrated the rib destruction/irregularity and the associated abscesses in our patients. USG showed abscesses as hypoechoic areas, with varying degrees of internal heterogeneity. Bone fragments appeared as echogenic foci within these hypoechoic collections. In our series, none of the patients had lesions localized to USG-inaccessible areas (such as the subscapular region) for which CT or MRI would have been necessary. Although there are no specific reports in the literature on MRI abnormalities in rib tuberculosis, one would expect altered bone marrow signal and enhancing soft tissue abscesses as reported in sternal and sterno-clavicular tuberculosis.[2,5]

The imaging modalities useful in chest wall tuberculosis involving the sternum, sternoclavicular joint, and ribs are radiography, USG, and CT scan, as highlighted in our report. The majority of earlier investigators have highlighted the role of CT scan only, and few workers have described MRI abnormalities.[2,3,5,7,11] Although MRI is a radiation-free modality with excellent delineation of soft tissue pathology, most orthopedicians in our institution primarily refer patients expected to have osteolytic/sclerotic lesions for a CT scan as the next modality after radiography. In our patients, the diagnosis was arrived at in all patients by radiography, USG, and CT scan, and confirmed by tissue aspirates/curettage. MRI was not deemed necessary in our patients. Recently, the role of PET/CT in the diagnosis of chest wall tubercular lesions has been discussed; these lesions are seen as areas of increased FDG uptake in active regions of granulomatous inflammation, with cold areas representing necrosed tissue.[12]

## Conclusion

Our report shows that possibility of tuberculous aetiology should be considered even for atypical sites of skeletal inflammation, such as sternum, sternoclavicular joint and ribs. The imaging features of chest wall tuberculosis using radiographs, CT scan, and USG have been discussed. The role of USG in evaluating cold abscesses associated with rib tuberculosis has been highlighted, along with the importance of CT and USG guided aspiration in confirming the aetiology.
